# Healthcare providers’ awareness and management of cardiovascular risks in women with hypertensive disorders of pregnancy and gestational diabetes

**DOI:** 10.1007/s00404-025-08012-8

**Published:** 2025-04-18

**Authors:** Sarah Halmer, Sophie Fohleitner, Franziska Jutz, Sascha Klee, Constance Busvine, Barbara Wichert-Schmitt, Susanne Schubert, Birgit Pfaller

**Affiliations:** 1 Department of Internal Medicine 1, University Hospital St. Pölten, Dunant-Platz 1, 3100 St. Pölten, Austria; 2 Karl Landsteiner University of Health Sciences, Dr. Karl-Dorrek-Straße 30, 3500 Krems, Austria; 3 Department of Gynaecology and Obstetrics, University Hospital St. Pölten, Dunant-Platz 1, 3100 St. Pölten, Austria; 4 Division of Biostatistics and Data Science, Karl Landsteiner University of Health Sciences, Dr. Karl-Dorrek-Straße 30, 3500 Krems, Austria; 5 Department of Cardiology and Medical Intensive Care, Kepler University Hospital, Krankenhausstraße 9, 4021 Linz, Austria

**Keywords:** Cardiovascular disease, Adverse pregnancy outcome, Preeclampsia, Postpartum care, Screening

## Abstract

**Introduction:**

Cardiovascular disease (CVD) is the leading cause of death and the mortality rate and prognosis of CVD in women are worse compared to men. Adverse Pregnancy Outcomes (APOs) are frequently overlooked sex-specific risk factors for CVD and affect up to one in five pregnant women. This study evaluated healthcare providers'(HCPs) awareness of the long-term cardiovascular risk associated with gestational diabetes mellitus and hypertensive disorders of pregnancy.

**Methods:**

A cross-sectional survey was conducted in Austria between March and August 2022 to assess HCPs’ knowledge, follow-up recommendations, and counseling regarding cardiovascular risk following APOs. The respondents were divided into general medicine, Obstetrics and Gynecology (O&G), general internal medicine, and cardiology.

**Results:**

Of the 175 responses, 20% (*n* = 35) were from general medicine, 39% (*n* = 68) from O&G, 39% (*n* = 69) from general internal medicine and cardiology, and 2% (*n* = 3) from other specialties. Although most respondents were aware of increased CVD risk following APOs, significant knowledge gaps were identified, particularly concerning the prevalence and timing of CVD onset after APOs. Over 50% do not counsel women with APOs on cardiovascular risk reduction strategies and approximately half do not counsel on the risk of recurrence of APOs. Less than 20% provide women with written follow-up information. Differences in expertise were observed among specialties, with O&G demonstrating the highest level of knowledge.

**Conclusion:**

This study identified knowledge gaps among HCPs in postpartum care for women with APOs highlighting the importance of standardized follow-up programs and the need for targeted education for HCPs.

**Graphical abstract:**

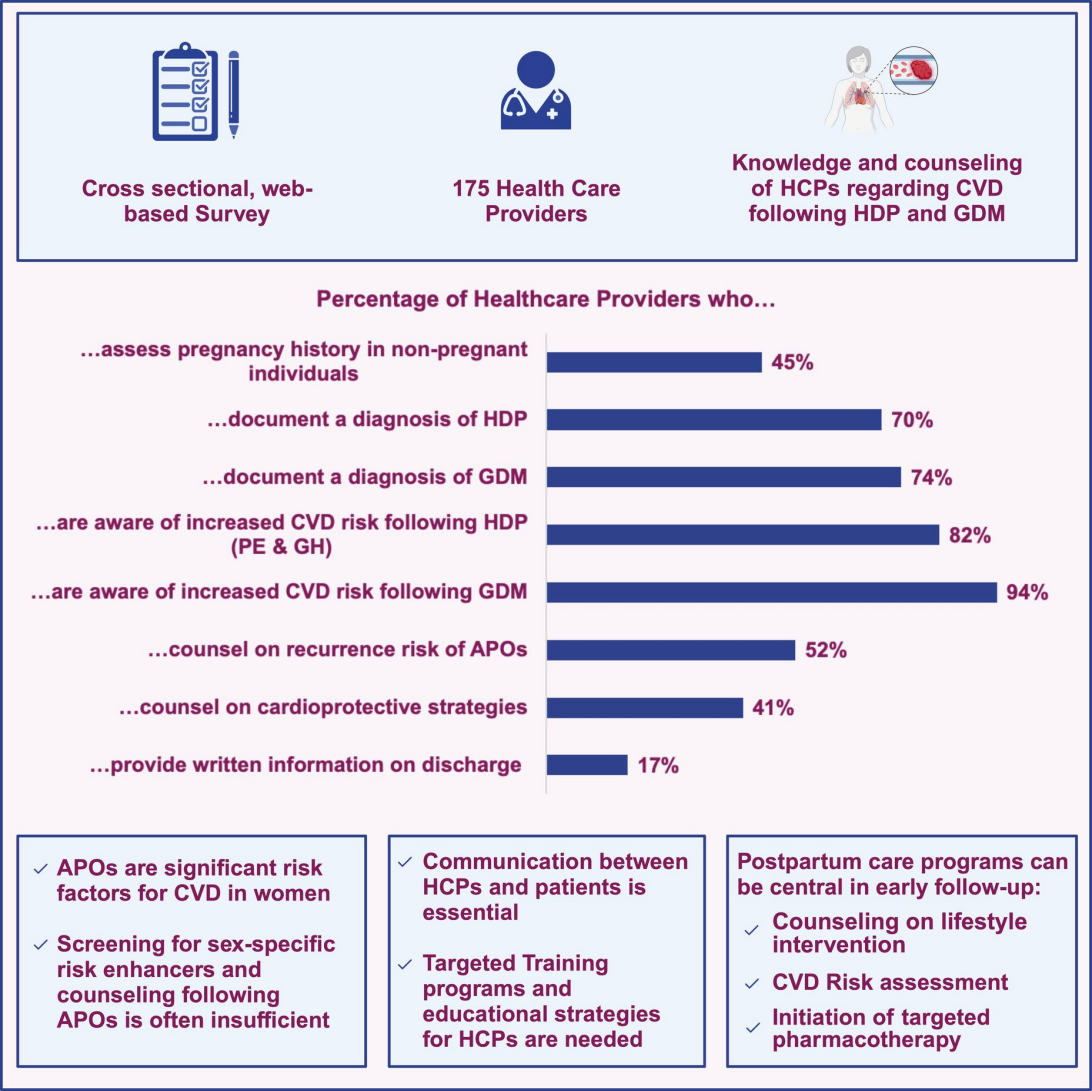

## What does this study add to the clinical work

**Table:** 

This study identified knowledge and care gaps among healthcare providers in the postpartum care of women with adverse pregnancy outcomes. Highlighting these gaps may advance standardization of postpartum care and improve development of training programs for healthcare providers.	

## Introduction

Cardiovascular disease (CVD) is the leading cause of death globally in men and women and has nearly doubled in the last three decades [1]. Although women have a lower prevalence of CVD, the mortality rate and prognosis are worse compared to men. Traditional risk factors of CVD are more associated with the male gender and women have been considered more protected; therefore, CVD risk is frequently underestimated, leading to underdiagnosis and undertreatment. Traditional risk factors like smoking, diabetes, hypertension, and obesity pose a higher relative risk for CVD in women compared to men [2]. Additionally, women are often underrepresented in cardiovascular clinical trials, contributing to inadequate management of cardiovascular risk factors and CVD and potentially affecting poorer outcomes [3]. Furthermore, sex-specific risk factors for women are frequently overlooked and include age of menarche and menopause, polycystic ovarian syndrome, pregnancy loss, and adverse pregnancy outcomes (APOs) [2].

In about 20% of pregnant women, underlying conditions, such as glucose intolerance, obesity, hypertension, and/or genetic predisposition, are present at the time of pregnancy and contribute to maladaptive mechanisms in pregnancy causing inflammation, endothelial dysfunction, and impaired hemodynamic adaptations, subsequently increasing the risk of APOs [4]. APOs include preeclampsia (PE), gestational hypertension (GH), gestational diabetes mellitus (GDM), intrauterine growth restriction (IUGR), small-for-gestational-age (SGA) delivery, placental abruption, and preterm delivery. In the long term, APOs raise the risk for chronic arterial hypertension, type 2 diabetes mellitus, metabolic syndrome, and CVD [5]. Women with GDM have an up to eight-times higher relative risk for developing DM, while PE infers an up to fourfold increased risk of heart failure and a twofold increase in coronary heart disease, stroke, and death [6–10]. The risk of chronic arterial hypertension is two-to-four times higher in women with HDP compared to those with normotensive pregnancies [9].

Early detection and screening for sex-specific cardiovascular risk factors in the postpartum period provides a critical opportunity to identify women at risk and help to initiate preventive measures to mitigate the risk for future CVD and chronic kidney disease (CKD) [11]. International societies including the International Society for the Study of Hypertension in Pregnancy, International Federation of Gynaecology and Obstetrics, and the European Society of Hypertension have updated recommendations regarding primary prevention and screening for CVD risk enhancers in women [11–16]. The 2019 guidelines on Primary Prevention of Cardiovascular Disease issued by the American College of Cardiology and the American Heart Association (AHA) advise healthcare professionals (HCPs) to evaluate for sex-specific CVD risk factors including APOs [17]. A more recently published AHA Scientific Statement on CVD Risk in the postpartum period emphasizes that high-risk individuals should receive intensified primary preventative measures and strongly argues for earlier and closer monitoring for the progression of CVD risk factors [6, 18].

Early intervention to reduce cardiovascular risks after APOs requires a multifaceted approach involving appropriate follow-up with HCPs, lifestyle intervention, and targeted pharmacotherapy [19]. However, there are limited data on HCPs’ knowledge and awareness of the association between APOs and CVD, and their adequate adherence to current guidelines [20].

This study assessed HCPs’ knowledge and routine practices regarding CVD risk assessment, counseling, and follow-up recommendations following HDP and GDM in Austria. Austria’s healthcare system provides universal public health insurance and prenatal checks are provided by obstetricians and general practitioners, including one routine assessment by an internal medicine specialist. Postpartum maternal assessments occur 6 weeks after delivery and are conducted by O&G specialists in the community. To our knowledge, this is the first study to evaluate differences in knowledge and counseling practices on long-term CVD risk following APOs across different specialties in this country.

## Methods

A cross-sectional survey was conducted in Austria between March and August 2022 using a web-based questionnaire to assess HCPs’ knowledge, follow-up recommendations, and counseling on APOs, particularly HDP and GDM. The respondents were divided into the specialties of general medicine, O&G, general internal medicine and cardiology, and others. Study participants were identified through an online search in the official list of physicians, from Austria’s largest physician search portal, and from the “Niederösterreichische Landesgesundheitsagentur (LGA)”. The survey was also shared on social media (Twitter, Facebook, and Instagram) to reach more physicians. To be eligible to participate, HCPs had to consent and actively practice in Austria, primarily in O&G, general medicine/family medicine, or general internal medicine. The extracted data were only used after the participant's consent was obtained. The total number of participants included was 175. A cross-sectional questionnaire consisting of 69 questions was composed in German language. The survey was accessible via a link created with Research Electronic Data Capture (REDCap) [21]. The main research objectives were to evaluate CVD risk assessment, diagnosis, counseling, and follow-up recommendations for women after HDP and GDM among HCPs in Austria. An ethics application (proposal number 1002/2022) was filed to the Karl Landsteiner University of Health Sciences.

### Statistical analysis

All data were analyzed using standard statistical packages (SPSS Version 28 for Windows, IBM Corp., Armonk, New York, USA). Data are presented descriptively in accordance with the data structure and data distribution. Results were compared between women diagnosed with HDP and women diagnosed with GDM. Chi-squared test or Fisher’s exact test was used to compare the subgroups (general medicine, O&G, general internal medicine and cardiology, and others) as appropriate.

## Results

In total, 266 HCPs answered the questionnaire, and 175 responses were included. The remaining 91 were excluded due to missing consent or incomplete response. Overall, there were slightly more female than male respondents. Of the 175 responses, 20% were from general medicine, 39% from O&G, 39% from general internal medicine and cardiology, and 2% from other specialties. Around two-thirds had more than 15 years of professional experience, with the largest group of experienced HCPs working in O&G. About one-third of the survey participants work in a non-hospital setting. Most HCPs regularly see women over the age of 50 and 32% report risk reduction counseling in this group of women. Further demographic details of the respondents are provided in Table 1. Around one-third counsel on cardiovascular risk reduction only in women with known CVD risk factors, and 11% hardly do so due to lack of time. Doctors working in general internal medicine and cardiology provide the most counseling regardless of known risk factors (52%).

**Table 1:** Baseline characteristics of respondents

	GM *n* (%)	O&G *n* (%)	IM&C *n* (%)	Others *n* (%)	Total *n* (%)
Total	35 (20)	68 (39)	69 (40)	3 (2)	175 (100)
*Sex*					
Female	23 (66)	44 (65)	26 (38)	3 (100)	96 (55)
Male	12 (34)	24 (35)	43 (62)	0 (0)	79 (45)
*Years of experience*					
< 5	5 (14)	9 (13)	15 (22)	1 (33)	30 (17)
5–10	7 (20)	11 (16)	19 (28)	0 (0)	37 (21)
> 15	23 (66)	48 (71)	35 (50)	2 (67)	108 (62)
*Workplace*					
Hospital	7 (20)	50 (74)	59 (86)	1 (33)	119 (68)
Non-hospital	28 (80)	18 (27)	10 (15)	0 (0)	56 (32)
*State of practice*					
Vienna	2 (6)	4 (6)	15 (22)	0 (0)	21 (12)
Lower Austria	24 (69)	45 (66)	36 (52)	3 (100)	108 (62)
Upper Austria	0 (0)	6 (9)	10 (15)	0 (0)	16 (9)
Salzburg	0 (0)	0 (0)	1 (1)	0 (0)	1 (1)
Carinthia	0 (0)	1 (2)	1 (1)	0 (0)	2 (1)
Styria	0 (0)	4 (6)	3 (4)	0 (0)	7 (4)
Tyrol	0 (0)	1 (2)	0 (0)	0 (0)	1 (1)
Vorarlberg	7 (20)	5 (7)	2 (3)	0 (0)	14 (8)
Burgenland	2 (6)	2 (3)	1 (1)	0 (0)	5 (3)
*Age of patients*					
18–35	4 (11)	30 (44)	4 (5)	1 (33)	39 (22)
36–50	7 (20)	32 (47)	9 (15)	1 (33)	49 (28)
> 51	24 (69)	6 (9)	56 (79)	1 (33)	87 (50)

GM, general medicine; O&G, obstetrics and gynecology; IM&C, internal medicine & cardiology

### Risk assessment and documentation of HCPs

Around one-third of HCPs counsel on cardiovascular risk reduction only in women with known CVD risk factors, and 11% hardly do so due to lack of time. Doctors working in general internal medicine and cardiology provide the most counseling regardless of known risk factors. More than two-thirds of HCPs evaluate and document a past history of HDPs (70%) and GDM (74%) in women, whereas less than half of all HCPs assess pregnancy history in non-pregnant individuals. Around one-third, 27%, replied that they never screen for sex-specific risk factors, including APOs. Respondents from O&G stated to record and document HDP significantly more often than the specialty of general medicine and general internal medicine and cardiology. O&G most frequently assessed for GDM with 85% stating always to document this diagnosis. In contrast, one-third of general internal medicine and cardiology stated to always assess for GDM. Detailed results are provided in Table 2.

**Table 2:** Screening for adverse pregnancy outcomes (HDP and GDM) by specialty

Specialty	Do you record and document HDP?			Do you record and document GDM?		
	Answers	Respondents *n* (%) of 175	*p* value (Fisher)	Answers	Respondents *n* (%) of 175	*p* value (Fisher)
GM (*n* = 35)	No	5 (14)	< 0.001	No	5 (14)	< 0.001
	Sometimes	8 (23)		Sometimes	5 (14)	
	Mostly	7 (20)		Mostly	8 (23)	
	Always	15 (43)		Always	17 (49)	
O&G (*n* = 68)	No	0 (0)		No	0 (0)	
	Sometimes	2 (3)		Sometimes	1 (2)	
	Mostly	7 (10)		Mostly	9 (13)	
	Always	59 (87)		Always	58 (85)	
GIM&C (*n* = 69)	No	19 (28)		No	14 (20)	
	Sometimes	18 (26)		Sometimes	20 (29)	
	Mostly	9 (13)		Mostly	12 (17)	
	Always	23 (33)		Always	23 (33)	
Others (*n* = 3)	No	0 (0)		No	0 (0)	
	Sometimes	1 (33)		Sometimes	1 (33)	
	Mostly	1 (33)		Mostly	2 (67)	
	Always	1 (33)		Always	0 (0)	

HDP, hypertensive disorder of pregnancy; GDM, gestational diabetes mellitus; GM, General medicine; O&G, obstetrics and gynecology; IM&C, internal medicine & cardiology

### Knowledge of HCPs regarding APOs

Most of the survey respondents, 63%, answered that they regularly treat women with HDP and 82% (*n* = 144) were aware that GH and PE both increase the risk of CVD. A small percentage, 3%, stated that only PE increased the risk for CVD in later life, and 13% answered only GH is a risk factor. However, 27% (*n* = 47) responded that they did not know the time window when CVD risk increases following a HDP and 32% (*n* = 56) knew that the cardiovascular risk manifests as early as 5–10 years following a pregnancy complicated by a hypertensive disorder. Regarding the prevalence of APOs 34% (*n* = 58) correctly answered that up to 20% of women suffer from APOs in general. Specialty-specific analysis demonstrated that 54% of general medicine providers assumed that 5% of all pregnant women suffer from APOs while less than 10% (*n* = 3) correctly estimated the prevalence to be 20% of all women (Table 3). While 83% (*n* = 151) knew about the increased risk for stroke or CVD following HDP, only 78% (*n* = 127) knew about the increased risk for CKD, and 70% (*n* = 122) for DM. Fewer than 10% of HCPs responded “I don’t know” when asked about the increased risk of stroke and CVD following HDP; however, the rates were higher for CKD and DM, 14% and 13%, respectively.

**Table 3:** Results of survey question: how many women suffer from adverse pregnancy outcomes?

	Total *n* (%) of 172	GM *n* (%)	O&G *n* (%)	IM&C *n* (%)	Others *n* (%)
5%	49 (29)	19 (54)	12 (18)	16 (24)	2 (67)
10%	65 (38)	13 (37)	25 (38)	26 (38)	1 (33)
Up to 20%	58 (34)	3 (9)	29 (44)	26 (38)	0 (0)

GM, General medicine; O&G, obstetrics and gynecology; IM&C, internal medicine & cardiology

The overall knowledge about long-term health risks following GDM was high with 94% (*n* = 164) being aware of increased CVD risk and 99% (*n* = 173) knowing about the risk of developing DM. Furthermore, 96% stated that DM is preventable after a diagnosis of GDM. While nearly all respondents knew of the increased risk for DM, only about half (55%) counseled on additional cardiovascular prevention measures such as self-blood pressure measurements following a diagnosis of GDM. Almost one-fifth (19%) of HCPs lacked awareness of the recommendation to repeat an oral glucose tolerance test (OGTT) within 12 weeks postpartum after being diagnosed with GDM. When asked about the rate of women performing an OGTT after delivery, most HCPs (70%) assumed that 25–50% of women undergo an OGTT within 4–12 weeks postpartum.

### HCPs care after hypertensive disorders of pregnancy and gestational diabetes

According to the results of the survey, most respondents provide postpartum care following HDP or GDM, including blood pressure measurement (97%), recording height and weight (91%), blood tests (91%), and offering follow-up recommendations (97%). However, more than half (59%) do not counsel women with APOs on cardioprotective strategies, and only 52% offer counseling regarding the recurrence of APOs (Table 4). Also, around half of the participants indicated that they provide follow-up recommendations after discharge from the hospital but only 17% (*n* = 27) of 159 respondents actively hand out written information. Over 80% of HCPs stated that they were not aware of written guidance on follow-up care for women with APOs. In this population, 128 HCPs (75%) recommend low-dose aspirin in a future pregnancy following APOs. A breakdown of the results showed that almost all obstetricians and 69% of general internal medicine and cardiology doctors recommended low-dose aspirin for future pregnancies, while only 44% of general medicine practitioners did so.

**Table 4:** Postpartum care recommendations after HDP or GDM

	No *n* (%)	Yes *n* (%)	Total *n* (%)
Blood-pressure measurements	6 (4)	166 (97)	172 (100)
Measurement of height and body weight	15 (9)	157 (91)	172 (100)
Blood screening	16 (9)	156 (91)	172 (100)
Follow-up recommendation	6 (4)	166 (97)	172 (100)
Recommendation for taking Thrombo-Ass in the subsequent pregnancy for women with high-risk, e.g., post-PE	43 (35)	128 (75)	171 (100)
Do you counsel patients with APOs and inform about cardioprotective strategies	101 (59)	71 (41)	172 (100)
Do your patients with APOs receive follow-up recommendations after discharge	77 (48)	83 (52)	160 (100)
Are you aware of written information for follow-up care of women with APOs & do you provide them for patients	132 (83)	27 (17)	159 (100)
Do you counsel patients regarding recurrence of APOs	77 (48)	83 (52)	160 (100)

HDP, hypertensive disorder of pregnancy; GDM, gestational diabetes mellitus; PE, preeclampsia; APO, adverse pregnancy outcome

### Postpartum care programs and postpartum mental health

In total, 71% (*n* = 111) of HCPs were aware of clinical programs covering postpartum care; however, only 29% (*n* = 46) stated that women received information about a follow-up program from the hospital. Among all HCPs, two-thirds stated that Internal Medicine would be the most suitable specialty to provide postpartum care follow-up following APOs, followed by 20% choosing O&G and 13% selecting general medicine. Nevertheless, 9% of O&G providers and 14% of general medicine providers reported never referring women with APOs to another medical specialty.

The survey results give an insight into the respondents’ knowledge regarding mental health disorders after childbirth, such as adjustment disorder, anxiety disorder, and depression. Of the 157 respondents, only 19% (*n* = 30) were aware that more than one in ten women suffer from mental health disorders after childbirth. Overall, 26% of the respondents stated that one in one hundred women suffer from mental health disorders after childbirth.

Finally, the participants were surveyed on their interest in education on long-term cardiovascular risks following APOs with 80% expressing an interest. Roughly three-quarters of HCPs stated that their preferred way to receive more information would be a webinar.

## Discussion

This study evaluated the knowledge and care practices of HCPs in Austria concerning cardiovascular risk in women with APOs, focusing on HDP and GDM. Our study demonstrated that screening for sex-specific risk enhancers for CVD is often insufficient despite good overall knowledge about CVD risk following APOs.

Over the last decades, intense research about sex-specific risk factors has taken place, since substantial evidence shows a link between APOs and future risk for CVD in women with APOs [6]. The postpartum period, often referred to as “fourth trimester”, is a critical period in a woman’s life to address potential CVD risk; HCPs play a crucial role in this time**.** The current knowledge and routine care of medical practitioners is therefore of considerable interest [18].

A scoping review by Roth et al. [20] is one of the most comprehensive review articles on this topic. We identified differences in knowledge levels between general internal medicine and cardiology, O&G, and general medicine providers with the latter exhibiting the least in-depth knowledge. Most HCPs in our study did not provide sufficient counseling on discharge, and the minority offered written follow-up recommendations.

### Risk assessment and knowledge gaps

Our results indicate that overall cardiovascular risk assessment related to APOs in non-pregnant women was low among HCPs and varied significantly between specialties, with O&G demonstrating higher levels of awareness. Detailed knowledge regarding prevalence of APOs and time of onset of risk increase was also low among our participants. This is in line with various similar recent studies. A Canadian research group surveyed Ontario-based obstetricians and 600 randomly selected GPs to assess their knowledge regarding HDP (GH and PE), and the associated CVD risk. Their analysis demonstrated poor overall knowledge, with only 54% and 64% correctly recognizing that PE and GH, respectively, increase future CVD risk. However, no comparisons were made between the responses of obstetricians and GPs [22].

A similar study conducted in Germany assessed O&G HCPs’ knowledge of CVD risk. They also evaluated counseling behavior and examined a correlation between knowledge of guidelines and risk counseling following PE [23]. Out of 212 obstetricians who completed a questionnaire, 86% were aware of the increased risk for hypertension and 79% of the increased risk of stroke. Only 78% correctly identified PE as a risk factor for CKD. Our research shows very similar results, indicating little overall awareness of the increased risk of CKD following HDPs among doctors [24]. Further on, they identified that only 45% of the respondents were familiar with the current German guidelines for follow-up after PE [25, 26]. It was established that physicians who knew the current guidelines were more likely to counsel women on self-BP measurement and long-term risk reduction [23].

Our findings on risk factor assessment and the higher rates of APOs documentation among O&G specialists align with a US-based survey, further signaling a greater overall awareness of PE as a risk factor in O&G professionals. This is likely associated with specialty training and their scope of practice compared to general medicine and general internal medicine and cardiology [20, 27]. However, the US survey also highlighted that once a history of APO was identified, internists were more knowledgeable on appropriate follow-up care than obstetricians. This included fasting glucose to screen for DM following PE or BP measurements and lipid panels following a diagnosis of GDM. Their analysis demonstrated a positive correlation between overall knowledge about CVD risk and the assessment of pregnancy history regardless of specialty [27].

Consistent with our findings, a study on follow-up after HDP and GDM indicated that internists and cardiologists were not sufficiently familiar with screening recommendations after a GDM diagnosis [28]. Furthermore, most respondents in our survey wrongly assumed that 25–50% of women undergo an OGTT postpartum. However, according to the literature, reported participation levels are as low as 14% [29].

A more recent analysis by Roth et al. [30] examined different knowledge levels in midwives, obstetricians, GPs, and cardiologists following GH and PE. Their results contrasted with several other studies, including ours, in that cardiologist had the highest overall knowledge scores. Supporting our findings, HCPs demonstrated limited knowledge about specific conditions, such as the risk of developing DM following PE and the early onset of CVD risk within the first 10 years after HDP. These gaps highlight potential areas for targeted education [30].

Limited awareness of short- and long-term complications following APOs also extends to mental health disorders: Only one-fifth knew that one in ten women suffer from mental health disorders following childbirth. Peripartum mental health disorders, including anxiety and depression, adversely affect both maternal and infant health outcomes. Women diagnosed with PE are at a significantly higher risk for postnatal depression and are more likely to describe childbirth as traumatic [31–33]. Beyond direct health impacts, mental health disorders are modifiable CVD risk factors [34, 35]. Simultaneously, they can be a barrier to care for CVD risk management and often need to be addressed before or alongside CVD risk factors [36].

### Counseling behavior and communication

Several studies focused on assessing the counseling practices of HCPs. Palmrich et al. [37] evaluated the awareness and postpartum recommendations of hospital-based O&G practitioners for women after PE and a more severe subtype of PE causing hemolysis, elevated liver enzymes, and low platelet count (HELLP-syndrome), in Austria. Their analysis showed a respectable compliance with guidelines in this selected group of highly specialized doctors: 97% advised women to continue BP measurement after a diagnosis of HDP and 100% consulted women on the risk of recurrence of HDP in subsequent pregnancies, further demonstrating that specialty training seems to be an enabling factor for adherence to guidelines [20, 37]. However, various other studies have found deficiencies in knowledge and application of guidelines on screening and follow-up recommendations [22, 24, 27, 38, 39]. Even when HCPs were educated about the link between APOs and CVD, further counseling and risk factor assessment were insufficient. Our study showed similar discrepancies between knowledge and its application with the minority of HCPs routinely advising women after APOs on cardioprotective strategies and only half of HCPs counseling on the recurrence risk for APOs. An additional concern is the communication of increased CVD risk from hospital-based HCPs to affected women and their primary care providers in the community. After hospital discharge, most women receive their routine follow-up in the community requiring a targeted transition of care between O&G or hospital internists to primary care providers, usually family medicine/GPs. This contrasts with the opinion of HCPs in our study, who consider that women are best followed up by internists. Yet less than one-fifth of our surveyed population pass on written follow-up information on discharge. MacDonald et al.’s [22] findings demonstrate a similar lack in communication of the increased long-term risk for hypertension to family physicians. Physicians report time and resource constraints, self-reported knowledge gaps, insufficient evidence-based research, and guideline inconsistencies as reasons for their lack of counseling [20, 40].

### Guidelines and education

The highlighted knowledge gaps underscore the need for education of HCPs about current guidelines on cardiovascular risk reduction in women. As identified in our study, HCPs expressed an interest in continuing professional development in the field of CVD risk following APOs. Access to multidisciplinary courses and training programs as well as evidence-based recommendations can guide HCPs in their postpartum care. Research shows that implementation of guidelines can help create awareness about CVD risk after APOs among medical professionals and women [20, 23, 36, 41]. Current guidelines recommend women and HCPs are informed about APOs and increased CVD risk; and cardiovascular risk screening and lifestyle counseling should take place 3 months postpartum [13, 15, 42]. The recently published guideline of the German Society of Gynecology and Obstetrics (AWMF) highlights the “fourth trimester” as a pivotal time to address complex long-term follow-up through a multidisciplinary effort involving obstetricians, physicians, and general practitioners [25]. Increasing the frequency and improving the content of communication at an early stage postpartum is deemed to be essential in conveying information to a high-risk population and their primary care providers to help mitigate women’s CVD risk [6, 19]. Standardized postpartum care programs and specialist clinics for mothers with APOs can be central in improving women’s long-term health and remote patient monitoring programs have shown to enhance patient engagement [43]. Structured follow-up programs in Canada and the US focusing on postpartum blood pressure control, CVD risk assessment, and counseling have shown to positively impact lifestyle changes and improve notoriously low follow-up rates of women [44–47]. An even more longitudinal view of lifetime follow-up as proposed by Parikh et al. [6] who recommend follow-up after 6 weeks, 12 weeks, 6 months, and then yearly following a transition from O&G to primary care might further optimize cardiovascular risk prevention in women.

### Strengths and limitations

Strengths of this study include a reasonably large sample size of 175 participants and a diverse participant pool of obstetricians, internists, and general practitioners. This study is the first of its kind in Austria and is therefore able to give valuable insight into an understudied field in this country. This study has several limitations. The respondents reflect a specific geographical region, which may not accurately represent nationwide practice. Compared to similar studies, midwives were omitted from the survey. Furthermore, study participants tend to agree with the questions and may also represent a more invested population than non-responders. This may lead to an overestimation of the surveyed knowledge and awareness levels. Another limitation is that it remains unclear why some participants did not counsel women on their CVD risk despite their awareness of it. A qualitative follow-up could have offered further insight.

## Conclusion

Adverse pregnancy outcomes, including HDP and GDM, are significant risk enhancers for CVD in women but are often underrepresented in risk assessments. The identified knowledge gaps among HCPs in our research contribute to disparities in care. Communication between women and HCPs, as well as between community-based and hospital-based HCPs, is essential in reducing the barriers to effective patient care. Delivering relevant information on risk reduction strategies to women and caregivers must be addressed in awareness campaigns and continuing medical education. Postpartum care programs can be pivotal in providing counseling on lifestyle intervention, risk assessment, and initiation of targeted pharmacotherapy early on following APOs, to use this window of opportunity for CVD prevention at a younger age. Furthermore, directing women to specialist clinics may act as motivation, encourage long-term follow-up, and translate into lifestyle changes [19, 20, 44]. Improving women’s long-term health by addressing CVD risk is ultimately not merely of the individual’s interest but a critical public health priority of the twenty-first century as CVD remains a leading cause of morbidity and mortality and a significant economic burden [48].

## Data Availability

All data and materials are available from the corresponding author (BP) upon reasonable request.

## Abbreviations

AHA: American Heart Association
APO: Adverse Pregnancy Outcome
CKD: Chronic kidney disease
CVD: Cardiovascular disease
DM: Diabetes mellitus
ESH: European Society of Hypertension
GDM: Gestational diabetes mellitus
GH: Gestational hypertension
HCP: Healthcare provider
HDP: Hypertensive disorders of pregnancy
ISSHP: International Society for the Study of Hypertension in Pregnancy
OGTT: Oral glucose tolerance test
O&G: Obstetrics and gynecology
PE: Preeclampsia
